# Acting in tandem

**DOI:** 10.7554/eLife.36489

**Published:** 2018-04-23

**Authors:** Robert A Battaglia, Ailong Ke

**Affiliations:** Department of Molecular Biology and GeneticsCornell UniversityIthacaUnited States

**Keywords:** aptamer, noncoding RNA, phosphoribosyl pyrophosphate, PRPP, gene regulation, *B. subtilis*

## Abstract

RNA structures called tandem riboswitches allow bacteria to employ complex logical operations in response to nutrient starvation.

**Related research article** Sherlock ME, Sudarsan N, Stav S, Breaker RR. 2018. Tandem riboswitches form a natural Boolean logic gate to control purine metabolism in bacteria. *eLife* **7**:e33908. doi: 10.7554/eLife.33908

According to the RNA World hypothesis, RNA was the original molecule from which all life has since evolved. Like DNA, RNA can store genetic information and replicate itself. However, it can also perform tasks that DNA leaves to proteins, such as catalyzing chemical reactions. Many of these roles rely on RNA molecules adopting sophisticated tertiary structures which are mostly conserved across the three kingdoms of life. This reflects the ancient origins of these structures, possibly in the RNA World.

In messenger RNA molecules in bacteria, certain sequences upstream of the coding region form structures called riboswitches that can bind various ligands. By stabilizing or altering these structures, the binding of the ligand turns the production of the downstream gene either on or off (hence the name riboswitch). A change in the concentration of a given ligand therefore regulates the production of a given protein via the relevant riboswitch. Most of these ligands are molecules that are essential for the cell to survive and grow, which suggests that riboswitches are ancient structures. Riboswitches are therefore of interest for at least two reasons: their role as metabolic regulators in bacteria and as potential relics of the RNA World.

A large number of putative riboswitches have been identified by analyzing bacterial genome sequences, but many of these have remained as 'orphans' because it has been difficult to identify the molecules they bind to (Weinberg et al., 2010; Weinberg et al., 2017). The large *ykkC* family of orphans can be subdivided in different classes and subclasses based on the tertiary structure of the riboswitches, which exact molecule they bind to, and which genes they control. Recently, the ligand shared by the most abundant *ykkC* classes was identified as guanidine. The remaining subclass, called ‘subtype 2’, has been shown to regulate a group of genes that is distinct from those regulated by other members of the *ykkC* family (Nelson et al., 2017). However, its ligand and exact role were still unclear.

Now, in eLife, Ronald Breaker and colleagues at Yale University – Madeline Sherlock as first author, Narasimhan Sudarsan and Shira Stav – report that there are four distinct classes of subtype 2 riboswitch (called 2a, 2b, 2c and 2d), and that they have identified the ligand that binds to the 2b riboswitch (Sherlock et al., 2018). This ligand is a molecule called PRPP (phosphoribosyl pyrophosphate), which has a central role in purine biosynthesis; in particular, it helps to make the nucleobases adenine and guanine, which are crucial building blocks for DNA and RNA. In a separate yet-to-be-published paper they report that the 2a riboswitch binds to ppGpp: this molecule is an example of an alarmone, a class of small signal molecules that are produced by bacteria when they are under stress (ME Sherlock, N Sudarsan, RR Breaker, In preparation).

Sherlock et al. go on to study the 2b riboswitch in more detail. On its own, this riboswitch appears to respond to high levels of PRPP by turning on genes that are involved in purine biosynthesis. However, the 2b riboswitch mostly works in tandem with another riboswitch that recognizes guanine, and the researchers studied how this 'tandem riboswitch' responded to different combinations of high and low levels of guanine and PRPP. They found that gene expression was suppressed when levels of guanine were high and levels of PRPP were low: however, genes were expressed for the three other possible combinations (Figure 1). The fact that the tandem riboswitch is acting like a logic gate is not itself unusual. However, the logic operation performed by the tandem riboswitch (the IMPLY operation) has not been observed in nature before.

**Figure 1.**
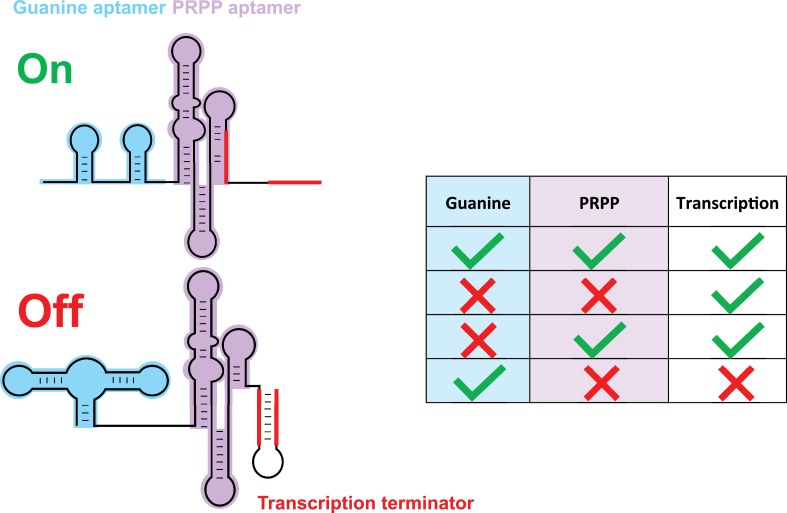
A tandem riboswitch as an IMPLY logic gate. Sherlock et al. studied a tandem riboswitch, made of a guanine riboswitch (blue) and a PRPP riboswitch (purple), that regulates genes involved in purine biosynthesis. These genes are transcribed when PRPP binds to its riboswitch (first and third rows in table) or when neither guanine nor PRPP are available (second row). However, a transcription terminator structure (red) forms and blocks transcription when PRPP is absent and guanine binds to its riboswitch (last row). This is the only known example of an IMPLY logic gate in nature. The PRPP riboswitch belongs to the larger family of *ykkC* riboswitches, which is subdivided in three main groups based on their tertiary structures. The riboswitches in this family bind to guanidine and participate in the guanidine detoxification process (Battaglia et al., 2017; Huang et al., 2017a; Huang et al., 2017b; Nelson et al., 2017; Reiss and Strobel, 2017; Reiss et al., 2017; Sherlock and Breaker, 2017; Sherlock et al., 2017).

Why would an organism evolve such a logic gate? When bacteria are deprived of nutrients, and the levels of both guanine and PRPP are low, one would expect purine biosynthesis to be reduced as the bacteria attempt to conserve their resources. However, somewhat counter-intuitively, the tandem riboswitch ensures that the genes responsible for purine biosynthesis continue to be expressed. Sherlock et al. reasoned that this might have something to do with the fact that bacteria need to keep their levels of GTP low and their levels of ATP high in response to starvation. GTP levels are kept low because this molecule prevents bacteria from producing their own amino acids, which is a necessary step during nutrient deprivation. However, ATP levels are kept high to promote the transcription of genes that are involved in the stress response to starvation. The guanine-PRPP riboswitch regulates genes involved in the first steps of purine biosynthesis or the shuttling of these precursors to ATP synthesis pathways. The end outcome is that the tandem riboswitch allows the genes necessary for ATP production to stay on during starvation without stimulating the synthesis of GTP.

In the tandem riboswitches reported to date, the individual structures typically operate independently of each other, with their outputs being combined later to establish a higher-order logic gate. However, the tandem riboswitches described by Sherlock et al. must depend on each other in some way. Further research is needed to understand this interdependency in greater detail.

The versatility of the *ykkC* class of riboswitches – at least five types of ligands can be recognized – surpasses anything that has been seen in the riboswitches that have been characterized to date. What is so special about this class of riboswitches and why is it involved in the regulation of so many different sets of genes? We look forward to researchers in bioinformatics and structural biology working together in the future to improve our understanding of the evolution of riboswitches.
